# Cannabis and Cognition: Connecting the Dots towards the Understanding of the Relationship

**DOI:** 10.3390/brainsci10030133

**Published:** 2020-02-27

**Authors:** Marco Colizzi, Sarah Tosato, Mirella Ruggeri

**Affiliations:** 1 Section of Psychiatry, Department of Neurosciences, Biomedicine and Movement Sciences, University of Verona, 37134 Verona, Italy; 2 Department of Psychosis Studies, Institute of Psychiatry, Psychology and Neuroscience, King’s College London, London SE5 8AF, UK

**Keywords:** delta-9-tetrahydrocannabinol, endocannabinoid system, executive functions

## Abstract

Several studies have advanced the understanding of the effects of cannabis on cognitive function. A comprehensive reappraisal of such literature may help in drawing conclusions about the potential risks associated with cannabis use. In summary, the evidence suggests that earlier age of use, high-frequency and high-potency cannabis use, as well as sustained use over time and use of synthetic cannabinoids, are all correlated with a higher likelihood of developing potentially severe and persistent executive function impairments. While the exact mechanisms underlying the adverse effects of cannabis on cognition are not completely clear, Magnetic Resonance Imaging (MRI) studies support the presence of both structural and functional alterations associated with cannabis use. Cognitive dysfunction is also a core feature of many neuropsychiatric disorders and care must be taken regarding the effects of cannabis use in these patient populations. Cognitive impairments affect patients’ daily functions, sociability, and long-term outcome, posing elevated economic, social, and clinical burdens. There is, thus, a compelling case for implementing behavioral and cognitive rehabilitation therapies for these patients, as well as investigating the endocannabinoid system in the development of new psychopharmacological treatments.

With around 200 million users worldwide, cannabis takes the lead when it comes to the number of people using a drug for recreational purposes [1]. The growing popularity of cannabis has seen a parallel increase of the public interest into its safety. Accumulating evidence associates cannabis use with several adverse behavioral, physiological, and neural effects [2], with acute challenge studies implying a causal relationship for such associations [3]. Indeed, studies of the long-term impact of cannabis suggest the development of tolerance [2] and dependence [4] upon sustained use. However, the harmful effects of cannabis are still debated, especially their severity and whether they are of a long-lasting nature. Interestingly, in a nine-category matrix of physical and social harm of both illicit and legal drugs, cannabis did not score in the top 10, while alcohol and tobacco did [5]. Cognitive function is one of the domains mostly investigated with reference to cannabis use, but also one of those generating the most conflicting results, with not all studies indicating poorer cognitive performance in otherwise healthy individuals or patients with a severe mental disorder and even some evidence of better performance in cannabis-using psychosis patients [6]. Studies of the effects of cannabis on cognition conducted over the last five decades have progressively unfolded a relationship of a complex nature, where several factors come into play. First, evidence indicates non-uniform disrupting effects of cannabis across different cognitive domains [7]. Second, genetic background may determine different individual susceptibility to cannabis-induced cognitive impairments [8,9]. Third, cognition seems to be the domain most likely to demonstrate tolerance upon repeated exposure, with some evidence of full tolerance indicating a complete absence of acute effects [2,10,11]. Fourth, cannabis composition and patterns of use play a relevant role, with both high-potency cannabis varieties, i.e., cannabis high in concentration of the psychoactive component delta-9-tetrahydrocannabinol (Δ9-THC) [12], and frequent cannabis use, e.g., daily [13], being associated with more pronounced cognitive impairments, thus supporting a cumulative adverse effect of Δ9-THC. Fifth, synthetic cannabinoids, which act as more potent full agonists at the cannabinoid receptor type 1 than Δ9-THC, thus exerting a more severe disruption of the endocannabinoid system, have been shown to induce more evident cognitive impairments in healthy subjects, which are undistinguishable from those observed in psychosis [14]. Finally, the use of cannabis in adolescence may lead to more serious cognitive impairments, due to the drug interfering with brain maturation [15]. 

An interesting up-to-date review article, “The Effects of Cannabinoids on Executive Functions: Evidence from Cannabis and Synthetic Cannabinoids—A Systematic Review”, published in *Brain Sciences*, brings together different lines of research about the effects of cannabis on cognition, including preclinical versus clinical evidence, acute versus long-term effects, occasional versus regular exposure and organic versus synthetic cannabinoids [16]. Such strategy emphasizes the importance of interpreting the available evidence altogether, to overcome the risks of interpreting the phenomenon based only on partial data [17]. Other merits of the review are that it applies rigorous inclusion criteria in terms of cognitive outcome measures, focusing only on objective measurements, as well as disentangles the effects of cannabis on each executive function sub-domain. High-level cognitive functions call on combinations of different component processes and there is evidence that changes in cognitive functioning, for instance, because of aging, are more likely to be masked when using more general cognitive measures compared to the use of more specific abilities [18]. It is, therefore, plausible that the same would happen with reference to the effects of cannabis use. Focusing on the three core executive functions, attention, working memory, and cognitive flexibility, separately [19], the authors make a noble attempt to deal with this potential issue. Moreover, in excluding studies performed on participants with psychiatric or substance use disorders, the review cut out two important arguments that could have hampered its conclusions; that is, the alternative explanation that the association between cannabis and cognitive impairments would be driven by use of other substances or coexisting psychopathological features, making cannabis users less proficient cognitively [20].

In the review by Cohen and Weinstein, one by one, all the apparent inconsistencies of the available literature find a possible explanation. Repeated exposure to cannabis is more clearly associated with the manifestation of executive function impairments. The evidence indicates a dose–response relationship for the effect of cannabis on executive functions, with frequent users and users of potent forms of cannabis presenting with more pronounced cognitive impairments. Exposure to synthetic cannabinoids is more clearly associated with long-lasting impairments. Exposure during adolescence increases the likelihood of such impairments being more severe and persisting in adulthood. 

The exact mechanisms underlying the adverse effects of cannabis on cognition are not completely clear. However, implementing studies of the effect of cannabinoids on cognition in a Magnetic Resonance Imaging (MRI) design may help understanding the underlying neurobiological mechanisms [6]. Consistently, the evidence from structural MRI studies reviewed here support an association between chronic cannabis use and reduced gray matter volumes in brain regions relevant to cognitive processes, including the hippocampus and amygdala, with the extent of such alterations correlating with age of onset, frequency, and severity of cannabis use. Similarly, functional MRI studies indicate disputed brain activity in regions involved in the processing of several cognitive tasks as a function of cannabis use. Interestingly, some of this evidence suggests that, while performing a cognitive task, cannabis users’ brain activity may be disrupted, even in the absence of a less proficient behavioral performance, reflecting an attempt to sustain performance by recruiting additional or different neural resources [21]. This would provide another possible explanation for the absence of the cannabis effect in those studies assessing exclusively the behavioral component of cognitive processing [22].

By affecting patients’ daily function, sociability, and long-term outcome, cognitive impairments place important socioeconomic burdens on society and patients themselves, also posing significant challenges to healthcare practitioners [23]. As Cohen and Weinstein point out, understanding how different cannabinoids may modulate cognitive processes can shed new light into the neurobiological mechanisms that increase the risk of long-lasting cognitive impairments in regular cannabis users. Moreover, cannabis use can increase the risk of developing disabling neuropsychiatric disorders, such as psychosis [24], and cognitive dysfunction is a core feature of such disorders [23]. Interestingly, endocannabinoid alterations have been implied in the pathophysiology of psychosis, independent of cannabis use [25]. Based on this evidence, along with the implementation of behavioral and cognitive rehabilitation therapies for these patients, there is also a compelling case for investigating the endocannabinoid system in the development of new psychopharmacological treatments [26].
